# Cortical thinning in young psychosis and bipolar patients correlate with common neurocognitive deficits

**DOI:** 10.1186/2194-7511-1-3

**Published:** 2013-04-17

**Authors:** Sean N Hatton, Jim Lagopoulos, Daniel F Hermens, Elizabeth Scott, Ian B Hickie, Maxwell R Bennett

**Affiliations:** Clinical Research Unit, Brain & Mind Research Institute, University of Sydney, 100 Mallet Street, Camperdown, New South Wales, 2050 Australia

**Keywords:** Psychosis, Bipolar, Cortical thinning, MRI, FreeSurfer, Neurocognition

## Abstract

**Background:**

People in midlife with established psychosis or bipolar disorder exhibit patterns of cortical thinning across several brain regions. It is unclear whether these patterns are indicative of a continuously active pathological process, residual effects of an earlier illness phase or pre-illness onset developmental risk factors. Here, we investigated whether cortical thinning is evident in younger patients in the early phase of psychosis or bipolar disorder and the relationship between cortical thinning and neurocognitive performance in young people.

**Methods:**

Magnetic resonance imaging was obtained from a sample of young patients with psychosis (*n* = 40; mean age 23.5 years), bipolar disorder (*n* = 73; mean age 21.9 years) or controls (*n* = 49; mean age 24.2 years). Group differences in cortical thickness were assessed using statistical difference maps, and regions of cortical thinning were correlated with medication dosage and performance on neurocognitive tasks. As initial comparisons using multiple corrections found no differences between the groups, follow-up analysis with a significance threshold of *p* < 0.001 was performed.

**Results and discussion:**

As distinct from reported findings in older subjects, young patients with psychosis have less extensive thinning in parietal-temporal areas and do not demonstrate significant thinning in the insula or dorsal lateral prefrontal cortex. Young patients with bipolar disorder exhibit cortical thinning in regions more consistent with those previously reported in paediatric bipolar patients. Although there were some differences in the regions of cortical thinning between the two groups, the shared regions of cortical thinning were correlated with neurocognitive deficits in visual sustained attention, semantic verbal fluency and verbal learning and memory that are commonly reported in young people with either psychosis or bipolar disorder.

**Electronic supplementary material:**

The online version of this article (doi:10.1186/2194-7511-1-3) contains supplementary material, which is available to authorized users.

## Background

While the majority of whole-brain morphometry studies use voxel-based morphometry (VBM) methods (Ashburner 2000), it is important to note that this technique is based on the change in the proportion of grey matter voxels. Consequently, the results are sensitive to smoothing filters and limit the achievable accuracy of complex three-dimensional cortical morphology. Cortical thickness, on the other hand, is determined by the size, density and arrangement of neurons, neuroglia, nerve fibres, blood vessels and extracellular space (Bennett 2011). The degree of cortical thinning may index underlying neuropathological changes such as myelin degradation, loss of dendrites and neurodegeneration (Benes and Francine 2003; Harrison 1999; Bennett 2011; Thompson et al. 2003). Therefore, these techniques have the potential to detect the signs of cortical changes that may be associated with the early stages of psychiatric illness (McGorry et al. 2006, 2009, 2010; Lagopoulos et al. 2012). To this end, examining changes in the pattern of cortical thickness in young patient populations, close to the period of illness onset, provides an opportunity to identify biological markers that are associated with illness onset or predictive of later trajectory.

Previous investigations of cortical thickness in young psychosis patients have provided mixed results (summarised in Table 1). When compared to healthy age-matched controls, there is significant cortical thinning within the frontal, temporal and parietal lobes in childhood- and adolescent-onset schizophrenia patients (White et al. 2003). Similar findings have been noted in first-episode psychosis patients (Narr et al. 2005a; Crespo-Facorro et al. 2011; Rais et al. 2010), and a follow-up study has highlighted additional cortical thinning within the occipital lobe in patients with little or no antipsychotic medication exposure (Narr et al. 2005b). Compared to subjects at ultra-high risk of psychosis who have not transitioned to a discreet psychotic disorder, schizophrenia patients exhibited cortical thinning within the frontal, temporal and precentral lobes, as well as the right insula region (Jung et al. 2011). These observations of cortical thinning in young patients with minimal medication exposure imply that these abnormalities may predate illness onset. This suggests that cortical morphometry may be used as a significant biomarker candidate for identifying early psychiatric disease states.

**Table 1:** **Overview of cortical thinning studies comparing young patients with psychosis, bipolar disorder and controls**

Author	Sample (age, years ± SD)	Regions of cortical thinning
White et al. (2003)	42 Childhood/adolescent-onset psychosis (17.7 ± 1.7)	• Mean cortical thickness
	24 Controls (17.7 ± 2.0)	• Frontal, temporal, and parietal sulci; temporal gyri
Narr et al. (2005a, b)	72 First episode psychosis (25.1 ± 4.7)	• Frontal, temporal and parietal lobes (significance set at *p* < 0.05, uncorrected)
	78 Controls (27.3 ± 6.6)	• Fronto-polar, occipital lobes in patients with little or no prior antipsychotic medication
Rais et al. (2010)	32 Early schizophrenia, non-cannabis users (23.3 ± 5.1)	• Same at baseline
	19 Early schizophrenia, cannabis users (21.8 ± 3.9)	• Five-year follow-up schizophrenia patients: right supplementary motor cortex, inferior frontal cortex, superior temporal gyrus, angular gyrus, cuneus and postcentral gyrus
	31 Controls (24.7 ± 6.7)	
Crespo-Facorro et al. (2011)	142 First episode psychosis (29.7 ± 8.7)	• Frontal, temporal and parietal lobes (group contrast only, not significant when covarying for gender)
	83 Controls (27.6 ± 7.6)	
Jung et al. (2011)	29 Ultra-high risk (UHR) of psychosis (22.2 ± 4.3)	• Mean cortical thickness: (controls = UHR) > schizophrenia
	31 Schizophrenia (24.3 ± 4.2)	• Schizophrenia vs controls: bilateral insular, inferior frontal, STG, PCC and ACC; left superior frontal, inferior temporal and precuneus; right parahippocampal, inferior parietal, lingual and precentral cortices
	29 Controls (23.2 ± 2.7)	
		• UHR vs controls: bilateral ACC and parahippocampal and medial frontal cortices; left STG; right lingual, inferior frontal, parietal and middle temporal cortices
		• Schizophrenia vs UHR: bilateral medial frontal cortex; left STG, superior frontal, parahippocampal and inferior temporal cortices; right insula, uncus, PCC and precentral and middle temporal cortices
Lyoo et al. (2006b)	25 Bipolar disorder (33.8 ± 9.6)	• Bilateral postcentral cortex; left DLPFC, ACC, PCC, occipital cortex; right orbitofrontal, angular and fusiform cortices
	21 Controls (31.5 ± 9.7)	• Bipolar I to bipolar II
Rimol et al. (2010, 2012)	173 Schizophrenia (32.3 ± 9.0)	• Schizophrenia vs controls: bilateral lateral and medial frontal lobe, temporal lobe, precuneus, parahippocampal and fusiform gyri, precentral gyrus, lateral and medial occipital lobe, lingual gyrus; left ACC, STG, middle temporal gyrus, inferior parietal and lingual gyrus; right medial orbitofrontal, entorhinal, supramarginal and inferior parietal cortices, isthmus of PCC
	139 Bipolar disorder (35.4 ± 11.3)	
	207 Controls (36.2 ± 9.7)	
		• Bipolar vs controls, schizophrenia: no significant findings
		• Bipolar I vs controls: bilateral lateral and medial frontal lobes; left orbitofrontal, posterior STG, inferior parietal gyrus; right superior frontal gyrus, supramarginal, parietal, inferior temporal and parahippocampal gyrus
		• Bipolar I vs schizophrenia: no significant findings
Foland-Ross et al. (2011)	34 Bipolar I disorder (38.1 ± 12.0)	• Bilateral prefrontal cortex; left ACC and dorsomedial, ventrolateral, frontopolar cortices
	31 Controls (37.8 ± 13.1)	• No difference between patients treated with or without lithium

ACC, anterior cingulate cortex; DLPFC, dorsolateral prefrontal cortex; PCC, posterior cingulate cortex; STG, superior temporal gyrus; UHR, ultra-high risk.

Hitherto there are only three studies that have investigated changes in cortical thickness in bipolar patients, and all have investigated middle-aged cohorts (summarised in Table 1). Compared to age-matched healthy controls, bipolar patients exhibit significant cortical thinning in the frontal, parietal and occipital lobes (Lyoo et al. 2006b) regardless of gender or medication exposure. A separate investigation has reported significant cortical thinning within the frontal, superior temporal and temporoparietal cortices of bipolar patients compared to healthy controls (Rimol et al. 2010, 2012). A recent study demonstrated that, in comparison to healthy controls, bipolar patients had cortical thinning within the bilateral prefrontal, dorsomedial, ventrolateral and frontopolar cortices, and this effect was not influenced by lithium dosage (Foland-Ross et al. 2011). Thus, while there is a general consensus on the regions of cortical thinning in bipolar disorder in middle-aged patients, questions remain regarding how these results relate to young patients who are typically closer to the onset of their disease process or how they compare with young patients who present with psychotic disorders.

Clarification of the cortical changes in young patients with psychosis or bipolar disorder is crucial for developing a better understanding of the pathological processes that underpin these disorders. This study assessed changes in cortical thickness among young patients with psychosis (schizophrenia, schizoaffective disorder, schizophreniform disorder and psychosis not otherwise specified) or bipolar disorder (bipolar I, bipolar II and bipolar spectrum disorder) and compared observed changes with age-matched control subjects. By comparison with the existing literature, our investigation examined whether cortical thinning occurred in the same regions of the brain in younger patients as are observed in older patients. Finally, we assessed how early cortical thinning relates to neurocognitive functioning in young people with a psychosis or bipolar disorder.

## Methods

### Subjects

One hundred and thirteen outpatients aged 16 to 30 years were recruited from specialist youth mental health clinics in Sydney, Australia (Scott et al. 2009, 2012). Forty-nine healthy control patients were recruited from the community in the same region and screened for any history of psychiatric disorders.

Exclusion criteria for both patients and controls were medical instability (as determined by a psychiatrist), history of neurological disease (e.g. tumour, head trauma, epilepsy), medical illness known to impact cognitive and brain function (e.g. cancer), intellectual and/or developmental disability, insufficient English for neuropsychological assessment and current substance dependence. All participants were asked to abstain from drug or alcohol use for 48 h prior to testing and informed about a drug screen protocol. The University of Sydney Ethics Committee approved the study. Participants gave written informed consent prior to participation in the study.

To determine the nature and history of any mental health problems, all subjects were assessed by a senior psychiatrist followed by further evaluation by a neuropsychologist using the Brain and Mind Research Institute Structured Interview for Neurobiological Studies (Scott et al. 2013). By consensus of the senior investigators (IBH and ES), subjects were assigned to diagnostic groups according to DSM-IV-TR criteria (American Psychiatric Association 2000). The psychosis group (*n* = 40) consisted of patients diagnosed with schizophreniform disorder (*n* = 20), schizophrenia (*n* = 10), schizoaffective (*n* = 4) or psychosis not otherwise specified (*n* = 6). The bipolar group (*n* = 73) consisted of patients diagnosed with bipolar I (BP1; *n* = 21), bipolar II (BP2; *n* = 29) or bipolar spectrum disorder (BPD; *n* = 23; as described in Angst (2007)). Patients diagnosed with a bipolar disorder with severe psychotic features were not eligible to be included in this study. At the time of assessment, 21% of patients were not taking any psychotropic medications; 33% were taking second-generation antidepressants, 49% were taking an atypical antipsychotic medication, 28% were taking a mood stabiliser (consisting of 8 subjects taking lithium and 20 taking anticonvulsants) and 3% were taking a stimulant. Of those medicated, 38% were taking more than one of these psychotropic medications; for the majority of these patients (35% of those medicated), this polytherapy included a second-generation antidepressant. A summary of medication by diagnostic grouping is provided in Table 2.

**Table 2:** **Medication category usage by diagnosis**

Diagnosis ***(n)***	Medication, ***n*** (%)				
	Unmedicated	Antidepressants	Antipsychotics	Mood stabilisers	Stimulants
***Psychosis group***					
Schizophreniform (20)	6 (24)	6 (24)	13 (52)	0 (0)	0 (0)
Schizophrenia (10)	2 (15)	2 (15)	8 (62)	1 (8)	0 (0)
Psychosis NOS (6)	0 (0)	3 (38)	3 (38)	2 (25)	0 (0)
Schizoaffective (4)	1 (17)	1 (17)	3 (50)	1 (17)	0 (0)
***Bipolar group***					
Bipolar I (21)	4 (13)	3 (9)	13 (41)	12 (38)	0 (0)
Bipolar II (29)	11 (26)	7 (16)	16 (37)	7 (16)	1 (2)
Bipolar spectrum (23)	5 (14)	11 (31)	12 (34)	5 (14)	1 (3)

Diagnosis was determined by DSM-IV-TR criteria (American Psychiatric Association 2000) with the exception of bipolar spectrum disorder characterised by Angst (2007). NOS, not otherwise specified.

### Clinical assessment

Premorbid intelligence (‘predicted IQ’) was estimated from the Wechsler Test of Adult Reading (Wechsler 2001). The assessment included the Hamilton Depression Rating Scale (HDRS, 17-item; Hamilton 1967) to quantify current (over the last 7 days) mood symptoms and the Brief Psychiatric Rating Scale (BPRS; Overall and Gorham 1962) to quantify current general psychiatric symptom severity. The 24-point BPRS total score is further subtyped by subscores assessing depression (somatic concern, anxiety, depression, suicidality, guilt, self-neglect), positive symptoms (hostility, grandiosity, suspiciousness, hallucinations, unusual thought content, bizarre behaviour, conceptual disorganization), negative symptoms (self-neglect, blunted affect, emotional withdrawal, motor retardation, uncooperativeness), mania (elated mood, conceptual disorganisation, tension, uncooperativeness, excitement, distractibility, motor hyperactivity) and disorientation (disorientation, mannerisms and posturing). Additionally, patients were assessed with the Young Mania Rating Scale (YMRS; Young et al. 1978), an 11-item diagnostic questionnaire use to measure the severity of manic episodes in paediatric patients over the previous 48 h.

### Magnetic resonance imaging acquisition and analysis

Participants underwent structural magnetic resonance imaging (MRI) scanning using a 3-T GE MR750 Discovery scanner (GE Medical Systems, Milwaukee, WI) at the Brain and Mind Research Institute, Camperdown, New South Wales, Australia. The images where acquired using a customized MP-RAGE 3D T1-weighted sequence to resolve anatomy at high resolution (0.9-mm isotropic resolution), TR = 7,264 ms, TE = 2,784 ms; pulse angle = 15, coronal orientation, FOV 230 mm^3^ and matrix of 256 × 256 × 196.

Cortical thickness and volumetric measurements were performed using the FreeSurfer software package version 5.1 (http://surfer.nmr.mgh.harvard.edu/), and technical details of these procedures have been previously described (Dale et al. 1999; Fischl and Dale 2000; Fischl et al. 1999a, b, 2001, 2002, 2004a, b; Han et al. 2006; Jovicich et al. 2006; Segonne et al. 2004). In brief, this process involved the following: motion correction and averaging of two volumetric T1-weighted images (Reuter et al. 2010), removal of non-brain tissue (Segonne et al. 2004), alignment of scans to the standard Talairach space, segmentation of the deep grey matter volumetric structures (Fischl et al. 2002, 2004a), intensity normalization (Sled et al. 1998), tessellation of the grey matter/white matter boundary, topology correction (Fischl et al. 2001; Segonne et al. 2007) and surface deformation to optimally place the grey/white and grey/cerebrospinal fluid borders (Dale et al. 1999; Dale and Sereno 1993; Fischl and Dale 2000). The subsequent cortical representations underwent surface inflation (Fischl et al. 1999a), registration to a spherical atlas to align individual cortical folding patterns with group cortical geometry (Fischl et al. 1999b), parcellation of the cerebral cortex into gyral and sulcal structures (Desikan et al. 2006; Fischl et al. 2004b) and creation of cortical thickness statistical maps, calculated as the closest distance from the grey/white boundary to the grey/CSF boundary at each vertex on the tessellated surface (Fischl and Dale 2000). FreeSurfer's procedure for automated measurement of cortical thickness has been validated against histological analysis (Rosas et al. 2002) and manual measurements (Salat et al. 2004) including in schizophrenic patients (Kuperberg et al. 2003).

Throughout the process, images were visually inspected, and any inaccuracies were manually edited. Statistical difference maps were smoothed using a 15-mm full width at half maximum Gaussian kernel with hemispheres analysed separately. Analysis comparing cortical thickness between cohorts covaried for age and gender. Initially, the significance threshold was set with a false discovery rate (FDR) of 0.05, and subsequent follow-up analysis set the significance threshold at *p* < 0.001 uncorrected (two-tailed), an approach that has been used in similar investigations (Lyoo et al. 2006b; Narr et al. 2005a, b). For ease of interpretation, only cortical thinning has been reported in contrasts between the psychosis and bipolar groups (i.e. cortical thinning in one cohort is cortical thickening in the other group and vice versa).

### Statistical analysis

Statistical analyses were performed using the Statistical Package for the Social Sciences (SPSS 20.0 for Mac). Intracranial volume outliers beyond a standard deviation of ±3.0 were removed from the analysis.

A *χ*^2^ test was used to compare categorical data, namely gender and handedness. One-way analyses of variance (ANOVAs) with follow-up Games-Howell Post Hoc analyses were used to assess differences in age, IQ, education and intracranial volume between psychosis, bipolar and control groups. Significance was set at *p* < 0.05 (two-tailed), and degrees of freedom were set at (2,159) with the exception of IQ (2,146).

Independent samples *t* tests examined the differences in age of illness onset, duration of illness, BPRS score and YMRS score between the psychosis and bipolar groups. Significance levels were set at *p* < 0.05 (two-tailed).

A follow-up comparison of subtypes of bipolar disorders examined differences in demographics and clinical somatology using a one-way ANOVA with follow-up Games-Howell Post Hoc analysis.

Significant regions of interest (ROIs) highlighted in the statistical difference maps were extracted as demarcated and described by the Destrieux cortical atlas (Destrieux et al. 2010). Cohen's *d* assessed the effect size of age-adjusted mean cortical thickness between groups. Partial correlation analysis was run to examine the association between psychotropic dosage and cortical thickness in these identified ROIs, controlling for gender and years of education (excluding control subjects).

To compare cortical thinning reported in older cohorts with our younger cohort, we used Cohen's *d* to examine differences between age-adjusted mean cortical thickness in ROIs highlighted in Rimol et al. (2010, 2012). These ROIs were defined by the Desikan-Killiany atlas (Desikan et al. 2006), where the inferior frontal gyrus comprised the lateral and medial orbitofrontal regions, the middle frontal gyrus comprised the rostral and caudal middle frontal regions, and the anterior cingulate cortex (Ant Cing) comprised the rostral anterior and caudal anterior cingulate.

### Neuropsychological assessment

To examine the implications of cortical changes to the neurocognitive performance in the psychosis and bipolar subjects, a trained research psychologist administered standardised tests as part of a broader battery (described previously) (Hermens et al. 2010a, b, 2011). The tests derived from the Cambridge Automated Neuropsychological Testing Battery (Sahakian and Owen 1992; Strauss et al. 2006) included the following: the rapid visual information processing task (RVP) to test visual sustained attention, Trail Making Test (TMT) to assess mental flexibility; the paired associate learning (PAL) to assess episodic memory and learning and the intra-dimensional/extra-dimensional task (IED) to test attention-set shifting. Verbal learning and verbal memory were assessed by the Rey Auditory Verbal Learning Test (RAVLT; Strauss et al. 2006), and verbal fluency was measured by the Controlled Oral Word Association Test (COWAT; Strauss et al. 2006). Age- and educational-adjusted *z*-scores were derived from normative data (Tombaugh et al. 1996). Control subjects were excluded from this analysis.

Neuropsychological scores beyond a standard deviation of ±3.0 were curtailed to values of +3.0 or −3.0 (depending on the direction), enabling a consistent range across variables as previously described (Naismith et al. 2002; Hermens et al. 2011). Partial correlation analysis examined the relationship between cortical thickness and either *z*-scores of neurocognitive performance or duration of illness covarying for years of education and gender. Spearman's rho correlation analysis examined the non-parametric distributions of the *z*-scores for the PAL and IED tests. The correlation analysis examined five groups: the BP1, BP2, BSD and psychosis groups to highlight diagnosis-specific neurocognitive deficits, and all subjects within the psychosis and bipolar groups collectively to highlight shared neurocognitive deficits.

## Results

### Demographic and clinical scores

Comparisons of demographic details between psychosis, bipolar and control groups revealed no significant difference in handedness, predicted IQ or intracranial volume (Table 3). The bipolar group was significantly younger (mean 21.9 years ± SD 3.6) than the control group (24.2 ± 2.7; *F*(2,159) = 7.31, *p* < 0.001), and the control group had more years of education (14.6 ± 2.1) than either the psychosis (12.6 ± 2.5) or bipolar groups (12.8 ± 2.1; *F*(2,159) = 12.61, *p* < 0.001).

**Table 3:** **Demographics and clinical scores (± standard deviation)**

	Psychosis ***(n =*** 40)	Bipolar ***(n =*** 73)	Controls ***(n =*** 49)	Significance test ***(p)***
Female, % (female/male)	27.5% (11/29)	71.2% (52/21)	57.1% (28/21)	*χ* ^2^ = 20.10 (<0.001)
Right handed, % (r/l/a)	85.0% (34/5/1)	80.8% (59/12/2)	83.7% (41/7/1)	*χ* ^2^ = 0.40 (0.982)
Age, years	23.5 ± 3.4	21.9 ± 3.6	24.2 ± 2.7	*F* = 7.31 (<0.001) bipolar < controls
Predicted IQ	101.9 ± 8.7	103.8 ± 7.9	104.9 ± 8.3	*F* = 1.31 (0.272)
Education, years	12.6 ± 2.5	12.8 ± 2.1	14.6 ± 2.1	*F* = 12.61 (<0.001) psychosis, bipolar < controls
Intracranial volume, cm^3^	1,557 ± 149	1,510 ± 127	1,532 ± 136	*F* = 1.57 (0.211)
Age of onset of illness, years	17.7 ± 4.3	14.9 ± 3.5	-	*t* = −3.75 (<0.001)
Duration of illness, years	5.8 ± 3.5	7.1 ± 3.9	-	*t* = 1.67 (0.097)
HDRS total	13.4 ± 6.8	12.4 ± 7.0	-	*t* = −0.75 (0.453)
BPRS total	44.6 ± 9.9	41.2 ± 8.9	-	*t* = −1.89 (0.062)
BPRS positive symptoms subscore^a^	13.8 ± 4.9	11.1 ± 3.6	-	*t* = −3.05 (0.003)
BPRS negative symptoms subscore	8.8 ± 3.7	6.7 ± 2.3	-	*t* = −3.14 (0.003)
BPRS depression subscore^a^	13.5 ± 4.4	14.2 ± 4.9	-	*t* = 0.71 (0.481)
BPRS mania subscore^a^	9.9 ± 3.3	10.8 ± 4.7	-	*t* = 1.16 (0.250)
BPRS disorientation subscore^a^	2.6 ± 1.2	2.2 ± 0.7	-	*t* = −2.02 (0.049)
YMRS total^a^	3.8 ± 8.1	11.8 ± 16	-	*t* = 3.66 (<0.001)

Significant differences in gender and handedness were evaluated using a Pearson Chi-square test. Age, predicted IQ and years of education were evaluated with a one-way ANOVA with Games-Howell Post Hoc analysis. All other significance values were evaluated using independent samples *t* tests. Significance levels were set at *p* < 0.05. ^a^Equal variance was not assumed. BPRS, Brief Psychiatric Rating Scale; HDRS, Hamilton Depression Rating Scale; YMRS, Young Mania Rating Scale.

Comparisons of clinical symptomology revealed differences between psychosis and bipolar groups (Table 3). The age of onset of the bipolar group (14.9 ± 3.5) was significantly less than that of the psychosis group (17.7 ± 4.3; *t*(111) = −3.81, *p* < 0.001), though the duration of illness between groups did not significantly differ (psychosis group 5.8 ± 3.5 years; bipolar group 7.1 ± 3.9 years; *t*(111) = 1.67, *p* = 0.097). While the patient groups did not significantly differ in clinical symptom severity (i.e. mean BPRS total score), compared to the bipolar group, the psychosis group indicated worse positive symptoms (respectively 11.1 ± 3.6; 13.8 ± 4.9; *t*(62) = −3.05, *p* = 0.003), negative symptoms (6.7 ± 2.3; 8.8 ± 3.7; *t*(56) = −3.14, *p* = 0.003) and disorientation (2.2 ± 0.7; 2.6 ± 1.2; *t*(53) = −2.02, *p* = 0.049). The YMRS total score indicated that the bipolar group reported a significantly worse manic symptom rating (11.8 ± 16.0) compared to the psychosis group (3.8 ± 8.1; *t*(102) = 3.66, *p* < 0.001).

In a follow-up analysis, the demographics and symptomology of subjects with BP1, BP2 or BSD were compared against those of control subjects (Table S1 in Additional file 1). Subjects with BP1 were younger than controls (respectively 21.5 ± 3.0; 24.2 ± 2.7; *F*(3,118) = 6.51, *p* < 0.001). Controls had more years of education (14.6 ± 2.1 years) than BP1 (12.4 ± 1.9) or BSD (12.3 ± 2.0; *F*(2,70) = 9.44, *p* < 0.001). Subjects with BSD had an earlier age of onset of illness (12.9 ± 3.2) than BP1 (16.3 ± 3.2) or BP2 (15.4 ± 3.2; *F*(2,70) = 6.98, *p* < 0.002). BP1 had a shorter duration of illness (16.3 ± 3.2 years) than BP2 (15.4 ± 3.2) or BSD (12.9 ± 3.2). Clinical symptomology was similar between groups with the exception of YMRS which indicated that subjects with BP1 had more severe mania symptoms (18.8 ± 20.1) than BSD (7.0 ± 12.0; *F*(2,70) = 3.36, *p* < 0.041).

### Differences in cortical thickness

**Figure 1:**
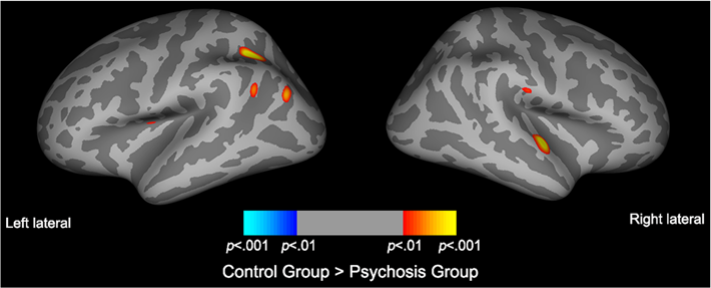
**Statistical maps of cortical thinning between psychotic and control subjects.** Statistical difference maps highlighting significant cortical thickness reductions between psychotic (*n* = 40) and control (*n* = 49) subjects accounting for gender and age identified several ROIs. Significance was set at *p* < 0.001 (uncorrected).

**Figure 2:**
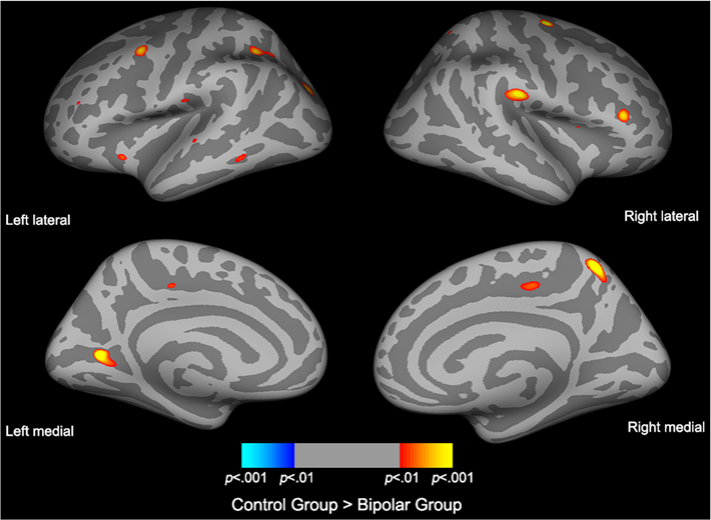
**Statistical maps of cortical thinning between bipolar and control subjects.** Statistical difference maps highlighting significant cortical thickness reductions between bipolar (*n* = 73) and control (*n* = 49) subjects accounting for gender and age identified several ROIs. Significance was set at *p* < 0.001 (uncorrected).

Initial analysis of cortical thickness differences between groups using FDR corrections gave no statically significant regions of cortical thinning. Adjusting the significance threshold to *p* < 0.001 (two-sided), statistical maps of cortical thickness differences between bipolar, psychosis and controls groups identified eight ROIs (Figures 1 and 2, Table 4, Table S2 in Additional file 1). The psychosis group showed cortical thinning predominantly in the left intraparietal sulcus and angular gyrus and right superior temporal gyrus compared to controls (Table 4, Figure 1). Conversely, the bipolar group showed cortical thinning predominantly in the left calcarine sulcus and right supramarginal gyrus, precuneus and precentral gyrus compared to controls (Table 4, Figure 2).

**Table 4:** **Mean cortical thickness and effect size within regions of interest in diagnostic groups**

	Mean thickness (mm)			Effect size ***(d)***		
	PSY	BPD	CON	CON-PSY	BPD-PSY	CON-BPD
**Left hemisphere**						
Intraparietal sulcus	2.03	2.06	2.02	−0.02	0.18	−0.19
Angular gyrus (pos.)	2.92	2.77	2.82	*−0.29*	*−0.43*	0.14
Angular gyrus (ant.)	2.46	2.61	2.48	0.06	*0.38*	*−0.31*
Calcarine sulcus	1.87	1.84	1.92	0.19	−0.12	*0.30*
**Right hemisphere**						
Superior temporal gyrus	2.9	2.83	2.9	−0.02	*−0.26*	*0.26*
Supramarginal gyrus	2.6	2.61	2.59	−0.02	0.06	−0.08
Precuneus	2.49	2.56	2.61	*0.42*	*0.24*	0.18
Precentral gyrus	2.31	2.36	2.33	0.07	0.19	−0.11
Fusiform	3.44	3.56	3.72	*0.59*	*0.28*	*0.35*
Parieto-occipital sulcus	2.89	2.93	2.94	0.10	0.08	0.01

Statistical difference maps of cortical thinning between psychosis (*n* = 40), bipolar (*n* = 73) and control (*n* = 49) groups accounting for gender and age identified in several regions of interest where *p* < 0.001 (uncorrected). Age-adjusted mean cortical thickness was obtained from FreeSurfer, and Cohen's *d* examined the difference between two means. Items of small- (d>0.2) or medium-effect size (d>0.5) are italicized. BPD, bipolar disorder group; PSY, psychosis group; CON, control group; ant., anterior; pos., posterior.

Contrasting the two patient cohorts (Table 4, Table S3 in Additional file 1), the psychosis group showed cortical thinning in the right fusiform compared to the bipolar group. Compared to the psychosis group, the bipolar group exhibited cortical thinning in the right parieto-occipital sulcus extending to the ventral posterior cingulate.

A recent investigation (Hegarty et al. 2012) showed that people with bipolar disorder had different patterns of cortical thinning between those with or without comorbid attention-deficit hyperactivity disorder (ADHD). Accordingly, a follow-up analysis which excluded three participants (BP2 *n* = 1; BSD *n* = 2) comorbid with ADHD found no statically significant differences in results (data not shown). No participants within the psychosis group had a secondary diagnosis of ADHD.

A follow-up analysis examined the differences in cortical thickness in these ROIs between bipolar subcategories. Compared to control subjects (Table 5), the small effect size of thinning of the right fusiform was consistent between BP1, BP2 and BSD subjects compared to control subjects. There was a medium effect size difference seen between BP1 and BSD subjects in the right supermarginal gyrus and right precuneus which was reflected in comparison with control subjects: BSD has a medium effect in the thickening of the right supermarginal gyrus, whereas BP1 subjects had a medium effect of thinning to the right precuneus. Compared to psychosis subjects (Table 6), BP1, BP2 and BSD subjects had a consistent medium size effect of thinning to the left posterior angular gyrus. The effect size of thinning to the right superior temporal gyrus was largest in BSD, reduced in BP2 and least in BP1 compared to the psychosis subjects.

**Table 5:** **Mean cortical thickness and effect size within regions of interest in bipolar subcategories vs controls**

	Mean thickness (mm)				Effect size ( ***d*** )					
	BP1	BP2	BSD	CON	CON-BP1	CON-BP2	CON-BSD	BP1-BP2	BP2-BSD	BP1-BSD
Left hemisphere										
Intraparietal sulcus	2.04	2.08	2.08	2.02	−0.07	*−0.29*	*−0.23*	−0.18	0.00	−0.15
Angular gyrus (pos.)	2.78	2.78	2.76	2.82	0.09	0.11	0.14	0.02	0.04	0.06
Angular gyrus (ant.)	2.64	2.64	2.56	2.47	*−0.47*	*−0.38*	*−0.20*	0.01	*0.20*	*0.25*
Calcarine sulcus	1.88	1.83	1.83	1.92	0.13	*0.34*	*0.37*	*0.20*	0.01	*0.23*
Right hemisphere										
Superior temporal gyrus	2.89	2.83	2.82	2.88	−0.02	0.19	*0.26*	0.17	0.05	*0.23*
Supramarginal gyrus	2.56	2.60	2.71	2.58	0.08	−0.07	*−0.51*	−0.15	−0.43	*−0.58*
Precuneus	2.47	2.59	2.61	2.61	*0.50*	0.09	0.01	*−0.39*	−0.08	*−0.50*
Precentral gyrus	2.33	2.37	2.37	2.33	−0.02	−0.13	−0.15	−0.11	−0.02	−0.14
Fusiform	3.56	3.58	3.58	3.71	*0.33*	*0.31*	*0.30*	−0.04	0.00	−0.04
Parieto-occipical sulcus	2.80	2.98	2.94	2.96	*0.29*	−0.05	0.05	*−0.34*	0.09	*−0.23*

Age-adjusted mean cortical thicknesses for ROIs were obtained from FreeSurfer, and Cohen's *d* examined the difference between people with bipolar I (*n* = 40), bipolar II (*n* = 29) or bipolar spectrum disorder (*n* = 23) and controls (*n* = 49). Items of small- (d>0.2) or medium-effect size (d>0.5) are italicized. ant., anterior; BP1, bipolar I disorder; BP2, bipolar II disorder; BSD, bipolar spectrum disorder; CON, controls; pos., posterior.

**Table 6:** **Mean cortical thickness and effect size within regions of interest in bipolar subcategories vs psychosis group**

	Mean thickness (mm)				Effect size ( ***d*** )		
	BP1	BP2	BSD	PSY	PSY-BP1	PSY-BP2	PSY-BSD
Left hemisphere							
Intraparietal sulcus	2.01	2.09	2.05	2.04	0.14	*−0.22*	−0.05
Angular gyrus (pos.)	2.77	2.78	2.74	2.96	*0.55*	*0.52*	*0.56*
Angular gyrus (ant.)	2.61	2.64	2.51	2.50	*−0.34*	*−0.34*	−0.04
Calcarine sulcus	1.89	1.83	1.82	1.88	−0.02	*0.21*	*0.30*
Right hemisphere							
Superior temporal gyrus	2.89	2.83	2.79	2.91	0.08	*0.28*	*0.46*
Supramarginal gyrus	2.57	2.61	2.70	2.58	0.04	−0.09	*−0.44*
Precuneus	2.46	2.58	2.58	2.53	*0.26*	−0.16	−0.15
Precentral gyrus	2.33	2.38	2.36	2.31	−0.04	*−0.23*	−0.17
Fusiform	3.53	3.59	3.57	3.45	−0.18	*−0.33*	*−0.27*
Parieto-occipical sulcus	2.84	2.97	2.96	2.91	0.14	−0.15	−0.10

Age-adjusted mean cortical thicknesses for ROIs were obtained from FreeSurfer, and Cohen's *d* examined the difference between people with bipolar I (*n* = 40), bipolar II (*n* = 29) or bipolar spectrum disorder (*n* = 23) and the psychosis group (*n* = 40). Items of small- (d>0.2) or medium-effect size (d>0.5) are italicized. ant., anterior; BP1, bipolar I disorder; BP2, bipolar II disorder; BSD, bipolar spectrum disorder; pos., posterior; PSY, psychosis group.

We performed a follow-up analysis of cortical thinning in regions previously identified in a cohort of older people with psychosis or bipolar disorder (Table S4 in Additional file 1). Our psychosis group had reduced cortical thickness in the bilateral inferior frontal gyrus compared to the bipolar or control groups (age-adjusted mean cortical thickness in the left inferior frontal gyrus, PSY 5.46 mm, BPD 5.64 mm, CON 5.59 mm; right inferior frontal gyrus, PSY 5.36 mm, BPD 5.55 mm, CON 5.54 mm). The bipolar group had reduced cortical thickness in the right superior parietal gyrus compared to the psychosis or control groups (age-adjusted mean cortical thickness PSY 2.37 mm, BPD 2.31 mm, CON 2.36 mm). Other regions investigated were of small or no significant effect size.

### Neurocognitive correlations

Correlation analysis across all subjects with psychosis and bipolar disorders found several neurocognitive domains that were associated with cortical thinning in these ROIs (Table 7). Of particular note, cortical thinning of the right supramarginal gyrus was correlated with worse performance in visual sustained attention (RVP mean latency, *r* = 0.30, *n* = 95, *p* = 0.004; RVP-A’, *r* = 0.21, *n* = 96, *p* = 0.038), semantic verbal fluency (COWAT Animals, *r* = 0.26, *n* = 98, *p* = 0.012) and verbal learning and verbal memory (RAVLT A7, *r* = 0.24, *n* = 101, *p* = 0.017). Duration of illness was not associated with any of the neurocognitive tasks analysed.

**Table 7:** **Interactions of cortical thinning or duration of illness with neurocognitive performance**

	Neurocognitive performance correlations ( ***r*** / ***rho*** )												
Regions of interest	RVP A’	RVP B”	RVP - mean latency	RAVLT - SUM	RAVLT - B1	RAVLT - A6	RAVLT - A7	COWAT - Letters	COWAT - Animals	TMT - A	TMT - B	PAL^a^	IED^a^
Left hemisphere													
Intraparietal sulcus	0.04	−0.15	*−0.23* ^*^	0.02	*0.23* ^*^	−0.02	−0.01	−0.01	−0.20	−0.13	−0.08	0.15	−0.05
Angular gyrus (pos.)	−0.04	*−0.24* ^*^	−0.12	0.09	0.09	0.04	0.07	0.01	0.00	−0.11	−0.06	0.05	−0.04
Angular gyrus (ant.)	0.16	−0.08	−0.02	0.03	0.07	0.07	0.1	0.09	0.04	−0.09	−0.08	0.12	−0.06
Calcarine sulcus	0.06	−0.02	0	0.17	0.03	*0.22* ^*^	0.19	0.08	0.03	0.09	0.15	0.04	0.01
Right hemisphere													
Superior temporal gyrus	0.1	0.02	0.1	0.07	−0.02	0.16	*0.20* ^*^	0.18	0.20	0.09	0.1	0.04	−0.15
Supramarginal gyrus	*0.22* ^*^	0.04	*0.30* ^**^	0.18	0.03	0.18	*0.24* ^*^	0.13	*0.26* ^*^	0.00	−0.03	0.16	−0.16
Precuneus	0.09	−0.07	−0.17	0.05	−0.01	0.03	0.07	−0.12	−0.16	−0.07	−0.02	−0.13	0.07
Precentral gyrus	−0.06	0.11	−0.16	−0.02	−0.07	0.01	0.04	−0.08	−0.15	−0.04	−0.09	−0.16	−0.09
Fusiform	0.2	−0.06	0.03	0.15	0.07	0.1	0.06	−0.1	−0.18	−0.04	0.07	0.15	0.18
Parieto-occipical sulcus	0.01	−0.13	−0.08	0.1	−0.05	0.11	0.1	−0.08	0.1	*−0.25* ^*^	−0.12	0.02	−0.16
Duration of illness	0.05	0.07	0.06	−0.09	0.04	−0.11	−0.09	0.07	0.02	0.02	−0.07	−0.12	−0.01

Partial correlation examined the relationship between either cortical thickness in regions of interest or duration of illness with *z*-scores of neurocognitive performance, controlling for gender and years of education, in all subjects with psychosis or bipolar disorder (n = 113). ^a^Spearman's rho correlation analysis examined non-parametric distributions of *z*-scores. Items of small-effect size (d>0.2) are italicized. *p < 0.05 (two-tailed), **p < 0.01 (two-tailed). ant., anterior; COWAT, Controlled Word Association Test; IED, intra/extra-dimensional shift; PAL, paired associate learning; RAVLT, Rey Auditory Verbal Learning Test; RVP, rapid visual processing; TMT, Trail Making Test; pos., posterior.

Correlation analysis across diagnostic groups found differing neurocognitive domains associated with cortical thinning in these ROIs (Tables S5, S6, S7 and S8 in Additional file 1). The BP1 group (Table S5 in Additional file 1) had several associations between cortical thinning in the left angular gyrus, right superior temporal gyrus and right supramarginal gyrus with neurocognitive deficits in visual sustained attention (RVP mean latency), verbal fluency (COWAT Letters, COWAT Animals) and verbal learning and memory (RAVLT Sum, RAVLT A6, RAVLT A7), although cortical thinning of the left anterior angular gyrus was also associated with better performance in the mental flexibility task (TMT B). The BP2 group (Table S6 in Additional file 1) had associations between cortical thinning of the right superior temporal gyrus and neurocognitive deficits in visual sustained attention (RVP B”), whereas cortical thinning of both the left posterior angular gyrus and right precuneus with improvements in verbal fluency (COWAT Letters), and cortical thinning of the right calcarine sulcus was associated with better performance in the episodic memory and learning task (PAL). The BSD group (Table S7 in Additional file 1) showed associations between cortical thinning of the left intraparietal sulcus and neurocognitive deficits in visual sustained attention (RVP mean latency), and the right parieto-occipital sulcus and episodic memory and learning (PAL), mental flexibility (IED) and cognitive processing speed (TMT A), whereas cortical thinning of the right precuneus was associated with better verbal fluency (COWAT). Finally, the psychosis group (Table S8 in Additional file 1) exhibited significant associations between cortical thinning of the right precentral gyrus and neurocognitive deficits in visual sustained attention (RVP mean latency) and mental flexibility (TMT B), while performance on the verbal fluency tests improved with cortical thinning to the left intraparital sulcus (COWAT Animals), right precentral gyrus (COWAT Letters, COWAT Animals) and right fusiform (COWAT Animals).

### Interactions with duration of illness and medications

A partial correlation analysis assessed the relationship between significant regions of cortical thinning and the daily dosage of psychotropic medication accounting for age and gender (Table S9 in Additional file 1). There was a correlation between increased mood stabiliser dosage and increased cortical thickness of the right precentral gyrus (*r* = 0.50, *n* = 28, *p* = 0.009; explains 25% of variance), as well as a correlation between increased antidepressant dosage and increased cortical thickness of the right fusiform (*r* = 0.38, *n* = 39, *p* = 0.019; explains 14% of variance). Duration of illness was negatively correlated with mood stabiliser dosage (*r* = −0.42, *n* = 24, *p* = 0.033; explains 18% of variance), suggesting that higher dosages of mood stabilisers were given at earlier stages of illness.

## Discussion

In contrast to reports of extensive cortical thinning of the temporal, frontal and insula regions in older patients with psychosis, we report that young patients with psychosis exhibit predominately parieto-temporal cortical thinning. Previous reports have provided evidence of parietal lobe abnormalities in psychosis, particularly the inferior parietal lobe encompassing the angular and supramarginal gyri (Shenton et al. 2001; Narr et al. 2005a, b; Schultz et al. 2010; Jung et al. 2011). Several investigations have reported structural abnormalities of the temporal cortex, particularly the superior temporal gyrus, primary auditory cortex and planum temporale in the left hemisphere in psychosis (Shenton et al. 2001; Kwon et al. 1999; Takahashi et al. 2007) that have given rise to auditory hallucinations or thought disorders (Barta et al. 1990; Shenton et al. 1992). Our present findings suggest that cortical thinning of the tempo-parietal regions is an early pathological hallmark in psychosis, consistent with other reports in young people with psychotic disorders (Narr et al. 2005a; Lyoo et al. 2011).

Our findings of cortical thinning in a group of young people with bipolar disorder show both similarities and differences with reports of cortical thinning in midlife bipolar disorder patients. In line with previous findings, our study found that young bipolar disorder patients have cortical thinning in the left calcarine sulcus (Lyoo et al. 2006b) and right supramarginal gyrus and superior parietal gyrus (Rimol et al. 2010, 2012). In contrast to these studies, we report that these young bipolar patients showed significant cortical thinning in the precuneus and superior aspect of the right precentral gyrus. A recent VBM study (Adleman et al. 2012) found that compared to aged-matched controls, paediatric bipolar patients (mean age 14.2 ± 2.6) exhibited grey matter reductions in the bilateral precuneus and the bilateral pre-supplementary motor area which is demarcated posteriorly by the precentral gyrus. Interestingly, in a 2-year follow-up, the precuneus grey matter volume of the paediatric bipolar group approached that of the control group though still remained significantly reduced. Collectively, the literature suggests that compared to healthy individuals, bipolar patients show degradation in different brain regions at various life stages (the precuneus and precentral gyrus in childhood; Adleman et al., 2012) which is also evident in young adulthood (present study), but not later in the illness course (Rimol et al. 2010, 2012; Lyoo et al. 2006b; Foland-Ross et al. 2011). This suggests that cortical thinning of the calcarine sulcus and supramarginal gyrus is an early life marker of bipolar disorder, whereas cortical thinning of the precuneus and precentral gyrus may be an early pathological event. Given the controversial nature of the diagnosis of paediatric bipolar disorder, the actual time course of these effects can only be determined in proper longitudinal studies. While it is possible that some effects resolve over time, it is also likely that quite different groups of subjects are actually being investigated.

Importantly, it was the common regions of cortical thinning between the psychosis and bipolar groups that produced significant correlations with neurocognitive deficits. Generally, these types of correlations suggest the functional significance of the MRI findings. Cortical thinning in the inferior parietal lobe, comprising the supramarginal gyrus and angular gyrus, and the adjacent intraparietal sulcus was strongly correlated with worse performance in visual sustained attention (RVP mean latency), semantic verbal fluency (COWAT Animals) and verbal learning and verbal memory (RAVLT A7). These results are consistent with the literature associating this region with language comprehension and decision-making (Hartwigsen et al. 2010), and cortical thinning of the parietal lobe has been associated with attention deficits in first-episode psychosis patients in contrast to healthy controls (Crespo-Facorro et al. 2011). Cortical thinning of the calcarine sulcus was also associated with verbal learning and verbal memory (RAVLT A6). The two diagnostic groups shared marginally significant cortical thinning of the anterior insula as we have previously demonstrated which is associated with attention-set shifting deficits (Hatton et al. 2012). Hence, it is likely that these shared regions of cortical thinning are functionally significant (and may impact on participation in employment or education) and contribute to those elements of cognitive dysfunction that are observed in young people with either psychosis or bipolar disorder (reviewed in Millan et al. 2012).

It is important to address our statistical approach of not using multiple corrections. Initial analysis of this data using FDR correction gave no statistically significant regions of cortical thinning. However, it is important to note that this present investigation contained subjects who had an early age of onset, a short duration of illness and less acute disorders (e.g. schizophreniform compared to schizophrenia, BSD or BP2 compared to BP1) compared to other studies on cortical thinning in these populations (summarised in Table 1). Furthermore, in the early stages of brain diseases during adolescence and early adulthood, changes are generally minimal and may lack regional specificity (Ashburner et al. 2003). Accordingly, we have used a moderately conservative statistical threshold of *p* < 0.001 for whole-brain analysis without multiple corrections to capture these subtle changes, and this approach has been employed in similar investigations (Lyoo et al. 2006b; Narr et al. 2005a, b). We have reported regions of cortical thinning below this threshold (0.01 > *p* > 0.001; Tables S2 and S3 in Additional file 1) to assist fellow researchers in subsequent analysis of these cohorts.

There are two additional limitations associated with our study that warrant discussion. Firstly, substance abuse in psychosis and bipolar disorder patients has been associated with changes in grey matter volumes that deviate from non-substance abuse psychosis (Lyoo et al. 2006a) and bipolar disorder (Jarvis et al. 2008) patients. The selection criteria for this study excluded substance dependence, but future research examining the relationship between the level of substance use and cortical thinning may delineate the role that comorbidity of substances has with respect to these potential biomarkers of these disorders. Secondly, the impact of pharmacological therapy on cortical lamina remains a contentious issue, with recent research suggesting that antipsychotic treatment may contribute to changes to frontal and temporal lobe cortical thickness (Smieskova et al. 2009; Navari and Dazzan 2009) while others have reported no effects (Kuperberg et al. 2003; Narr et al. 2005a; Nesvag et al. 2008). The correlation between increased mood stabiliser dosage and increased cortical thickness of the right precentral gyrus (25% of variance) should be treated with caution given the small number of patients on this treatment (*n* = 28; 8 subjects were taking lithium and the remaining 20 subjects were taking anticonvulsants). This study was cross-sectional in design, so no clear picture can be gained about the ways in which medication influences cortical thinning over time within the same individuals. The apparent differences between different reported age groups for bipolar disorder or psychosis may reflect other factors including different selection criteria, different diagnostic thresholds and concurrent exposure to other factors such as alcohol or substance misuse or medications.

## Conclusions

This investigation shows that cortical thinning of the tempo-parietal regions is present early in young patients with psychosis. This finding is in contrast to reported evidence of extensive cortical thinning to the temporal, frontal and insula regions in older patients with psychosis. Young patients with bipolar disorder exhibit novel cortical thinning patterns, more like those observed in paediatric as distinct from older bipolar patients. While there are some differences in the patterns of cortical thinning between the young psychotic and bipolar disorder groups, those regions of cortical thinning that are shared are those that were associated with the type of cognitive dysfunction that is typically observed in young people with either psychosis or bipolar disorder. While psychotic and bipolar disorders may have differing neuropathological origins, it is the shared regions of cortical thinning that underpin those observed neurocognitive deficits that impact on the lives of young people with psychosis or bipolar disorder.

## Electronic supplementary material

Additional file 1: **Supplementary tables.** A document showing nine supplementary tables. (DOCX 209 KB)

## Abbreviations

ACC: Anterior cingulate cortex
ADHD: Attention-deficit hyperactivity disorder
ANOVA: Analysis of variance
BP1: Bipolar I disorder
BP2: Bipolar II disorder
BPRS: Brief Psychiatric Rating Scale
BSD: Bipolar spectrum disorder
COWAT: Controlled Oral Word Association Test
DLPFC: Dorsolateral prefrontal cortex
FDR: False discovery rate
HDRS: Hamilton Depression Rating Scale
IED: Intra-dimensional/extra-dimensional
MRI: Magnetic resonance imaging
PAL: Paired associate learning
PCC: Posterior cingulate cortex
RAVLT: Rey Auditory Verbal Learning Test
ROI: Region of interest
RVP: Rapid visual information processing task
STG: Superior temporal gyrus
TMT: Trail Making Test
VBM: Voxel-based morphometry
UHR: Ultra-high risk
YMRS: Young Mania Rating Scale.
