# Supplementary material for: Cortical thinning in young psychosis and bipolar patients correlate with common neurocognitive deficits
Source: Int J Bipolar Disord. 2013 Apr 17;1:3. doi: 10.1186/2194-7511-1-3 (PMC4230309; doi:10.1186/2194-7511-1-3)
Supplement: Supplementary file 1 — Additional file 1: Supplementary tables. A document showing nine supplementary tables. (DOCX 209 KB) [file 40345_2012_3_MOESM1_ESM.docx]

# Additional File 1

|  | **Demographics and clinical scores (± standard deviation)** | | | | |
| --- | --- | --- | --- | --- | --- |
|  | **BP1 (*n* = 21)** | **BP2 (*n* = 29)** | **BSD (*n* = 23)** | **CON (*n* = 49)** | **Significance test [*p*]** |
| **Female, % (female/male)** | 52.4% (11/10) | 79.3% (23/6) | 78.3% (18/5) | 57.1% (28/21) | χ^2^ = 7.23 [.065] |
| **Right handed, % (r/l/a)** | 76.2% (16/5/0) | 86.2% (25/3/1) | 78.3% (18/4/1) | 83.7% (41/7/1) | χ^2^  = 0.40 [.982] |
| **Age, years** | 21.5 ± 3.0 | 23.0 ± 3.8 | 21.0 ± 3.7 | 24.2 ± 2.7 | *F* = 6.51 [<.001]  BP1 < CON ^*^ |
| **Predicted IQ** | 104.1 ± 7.5 | 105.9 ± 6.4 | 100.9 ± 9.2 | 104.9 ± 8.3 | *F* = 1.85 [.143] |
| **Education, years** | 12.4 ± 1.9 | 13.5 ± 2.0 | 12.3 ± 2.0 | 14.6 ± 2.1 | *F* = 9.44 [<.001]  BP1, BSD < CON ^*^ |
| **Intracranial volume, cm^3^** | 1532 ± 154 | 1494 ± 102 | 1510 ± 131 | 1532 ± 136 | *F* = 0.62 [.602] |
| **Age of onset of illness, years** | 16.3 ± 3.2 | 15.4 ± 3.2 | 12.9 ± 3.2 | - | *F* = 6.98 [.002]  BSD < BP1, BP2 ^*^ |
| **Duration of illness, years** | 5.2 ± 3.0 | 7.6 ± 3.7 | 8.1 ± 4.3 | - | *F* = 3.66 [.031]  BP1 < BP2, BSD ^*^ |
| **HDRS total** | 11.8 ± 7.0 | 13.67± 7.1 | 11.2 ± 6.9 |  | *F* = 0.89 [.416] |
| **BPRS total** | 41.6 ± 10.5 | 41.6 ± 8.0 | 40.2 ± 8.6 | - | *F* = 0.187 [.830] |
| **BPRS Positive Symptoms subscore^#^** | 11.8 ± 4.4 | 10.9 ± 3.1 | 10.7 ± 3.3 | - | *F* = 0.54 [.587] |
| **BPRS Negative Symptoms subscore** | 6.6 ± 2.2 | 6.8 ± 2.6 | 6.7 ± 1.9 | - | *F* = 0.08 [.927] |
| **BPRS Depression subscore^#^** | 13.0 ± 4.6 | 14.9 ± 5.2 | 14.3 ± 4.8 | - | *F* = 0.93 [.400] |
| **BPRS Mania subscore^#^** | 11.8 ± 5.8 | 10.7 ± 4.7 | 10.0 ± 3.6 | - | *F* = 0.80 [.453] |
| **BPRS Disorientation subscore^#^** | 2.3 ± 1.0 | 2.1 ± 0.3 | 2.3 ± 0.7 | - | *F* = 1.22 [.301] |
| **YMRS total^#^** | 18.8 ± 20.1 | 10.5 ± 14.0 | 7.0 ± 12.0 | - | *F* = 3.36 [.041]  BSD < BP1 ^*^ |

**Table 1: Demographics and clinical scores by bipolar subcategories.** Significant differences in gender and handedness were evaluated using a Pearson Chi-square test. All other significance values were evaluated using a one-way ANOVA with Games-Howell Post Hoc analysis. ^*^ Significance levels were set at *p* < .05. BP1, bipolar I disorder; BP2, bipolar II disorder; BPRS: Brief Psychiatric Rating Scale; BSD, bipolar spectrum disorder; CON, controls; HRSD, Hamilton Rating Scale for Depression; YMRS, Young Mania Rating Scale.

|  |  |  | **Talairach coordinates** | | |
| --- | --- | --- | --- | --- | --- |
| **Region of interest** | **Number  of  voxels** | **Area (mm^2^)** | ***x*** | ***y*** | ***z*** |
| **Psychosis vs Controls**  ***Left hemisphere*** |  |  |  |  |  |
| Intraparietal sulcus ^**^ | 758 | 277.7 | -29.8 | -50.7 | 34.7 |
| Angular gyrus (pos.) ^**^ | 222 | 129.9 | -44.2 | -72.6 | 25.9 |
| Angular gyrus (ant.) ^**^ | 172 | 83.7 | -47.8 | -56.9 | 25 |
| Paracentral lobe | 151 | 60.6 | -7.8 | -25.9 | 61.1 |
| Calcarine sulcus | 98 | 37.5 | -25.1 | -69.2 | 5.6 |
| Insula (ant.) | 44 | 13.8 | -35.7 | -3.9 | 16.5 |
| ***Right hemisphere*** |  |  |  |  |  |
| Superior temporal gyrus ^**^ | 569 | 262.9 | 53.1 | -9.4 | -1.2 |
| Supramarginal gyrus | 91 | 34.1 | 58.9 | -20.2 | 21.2 |
| Precuneus | 52 | 21.8 | 7.1 | -61.9 | 51.7 |
| **Bipolar vs Controls**  ***Left hemisphere*** |  |  |  |  |  |
| Calcarine sulcus ^**^ | 608 | 277.5 | -25.2 | -69.5 | 3.3 |
| Intraparietal sulcus | 473 | 165.9 | -29.5 | -50.7 | 35.1 |
| Transverse occipital sulcus | 227 | 113.4 | -28.6 | -77.5 | 14.4 |
| Middle frontal gyrus (cau.) | 216 | 127.1 | -39.5 | 5.6 | 48.3 |
| Insula (ant.) | 103 | 47.1 | -32.7 | 16.1 | -6 |
| Middle temporal sulcus | 71 | 46.6 | -55.6 | -45.7 | -10.6 |
| Dorsal posterior cingulate | 51 | 17.9 | -14.7 | -27.2 | 36.2 |
| Subcentral gyrus | 50 | 22.4 | -56.1 | -17.7 | 17.7 |
| Superior temporal gyrus | 37 | 13.6 | -63.2 | -17.8 | -0.6 |
| Middle frontal gyrus (ros.) | 12 | 7.8 | -39.3 | 37.3 | 18.7 |
| ***Right hemisphere*** |  |  |  |  |  |
| Supramarginal gyrus ^**^ | 602 | 242.6 | 52.6 | -24.6 | 23.8 |
| Precuneus ^**^ | 478 | 179.7 | 8.9 | -56.3 | 55.7 |
| Precentral sulcus ^**^ | 283 | 125.9 | 22.8 | -12.3 | 56.6 |
| Pars triangularis | 200 | 113.9 | 45.4 | 30.1 | 11.7 |
| Cingulate sulcus | 172 | 53.0 | 16.8 | -24.5 | 39.8 |
| Superior parietal lobe | 30 | 13.9 | 19.8 | -60.6 | 59.7 |
| Pars opercularis | 10 | 3.1 | 35.8 | 10.4 | 13 |

**Table 2. Cortical thinning between psychosis, bipolar and control groups.** Statistical difference maps of cortical thinning between the psychosis group (*n* = 40), bipolar group (*n* = 73) and control (*n* = 49) subjects accounting for gender and age identified several regions of interest where *p* < .01 (uncorrected). ** Significance was set at *p* < .001 (uncorrected). ant., anterior; cau., caudal; pos., posterior; ros., rostal.

|  |  |  | **Talairach coordinates** | | |
| --- | --- | --- | --- | --- | --- |
| **Region of interest** | **Number of voxels** | **Area (mm^2^)** | ***x*** | ***y*** | ***z*** |
| **Psychosis vs Bipolar**  ***Left hemisphere*** |  |  |  |  |  |
| No significant thinning |  |  |  |  |  |
| ***Right hemisphere*** |  |  |  |  |  |
| Fusiform ^**^ | 269 | 146.6 | 37.5 | -41.7 | -21.6 |
| **Bipolar vs Psychosis**  ***Left hemisphere*** |  |  |  |  |  |
| Precentral gyrus | 410 | 182.0 | -56.6 | -2.2 | 17.9 |
| Precuneus | 111 | 44.1 | -7.9 | -53.9 | 56.3 |
| Superior frontal gyrus | 100 | 58.7 | -7.2 | 47.3 | 36.3 |
| Superior parietal lobe | 97 | 33.5 | -16.0 | -44.7 | 72.6 |
| Superior parietal lobe | 73 | 31.1 | -13.1 | -67.2 | 58.8 |
| Inferior temporal gyrus | 52 | 31.3 | -53.0 | -27.5 | -29.9 |
| Superior parietal lobe | 48 | 18.6 | -24.3 | -59.5 | 61.2 |
| Anterior cingulate (ven.) | 20 | 9.1 | -3.7 | 20 | -4.1 |
| ***Right hemisphere*** |  |  |  |  |  |
| Parieto-occipical sulcus ^**^ | 441 | 213.4 | 5.7 | 25.6 | -9 |

**Table 3. Cortical thinning between psychosis and bipolar groups.** Statistical difference maps of cortical thinning in the psychosis group (*n* = 40) compared to the bipolar group (*n* = 73) accounting for gender and age identified several regions of interest where *p* < .01 (uncorrected). ** Significance was set at *p* < .001 (uncorrected). For ease of interpretation, only cortical thinning was reported. ven., ventral.

|  | **Left Hemisphere** | | | | | | |  | **Right Hemisphere** | | | | | | |
| --- | --- | --- | --- | --- | --- | --- | --- | --- | --- | --- | --- | --- | --- | --- | --- |
|  | Mean thickness (mm) | | |  | Effect Size (*d*) | | |  | Mean thickness (mm) | | |  | Effect Size (*d*) | | |
|  | PSY | BPD | CON |  | CON-PSY | BPD-PSY | CON-BPD |  | PSY | BPD | CON |  | CON-PSY | BPD-PSY | CON-BPD |
| IFG | 5.46 | 5.64 | 5.59 |  | **0.46** | **0.60** | -0.17 |  | 5.36 | 5.55 | 5.54 |  | **0.69** | **0.63** | -0.02 |
| MFG | 5.27 | 5.25 | 5.30 |  | 0.13 | -0.05 | 0.19 |  | 5.04 | 5.02 | 5.01 |  | -0.12 | -0.05 | -0.05 |
| SFG | 2.95 | 2.96 | 2.99 |  | **0.23** | 0.05 | 0.18 |  | 2.87 | 2.88 | 2.91 |  | **0.25** | 0.05 | 0.19 |
| ACC | 5.80 | 5.90 | 5.93 |  | **0.32** | **0.23** | 0.07 |  | 5.67 | 5.77 | 5.77 |  | **0.23** | **0.21** | 0.00 |
| MTG | 2.88 | 2.88 | 2.85 |  | -0.18 | 0.00 | -0.18 |  | 2.85 | 2.82 | 2.86 |  | 0.04 | -0.12 | 0.17 |
| STG | 2.82 | 2.77 | 2.79 |  | -0.19 | **-0.26** | 0.10 |  | 2.73 | 2.70 | 2.73 |  | -0.01 | **-0.22** | **0.22** |
| LatOccip | 2.23 | 2.21 | 2.24 |  | 0.03 | -0.15 | 0.18 |  | 2.41 | 2.40 | 2.44 |  | **0.23** | -0.07 | **0.29** |
| SPG | 2.29 | 2.27 | 2.28 |  | -0.09 | **-0.21** | 0.13 |  | 2.37 | 2.31 | 2.36 |  | -0.15 | **-0.48** | **0.37** |

**Table 4. Mean cortical thickness and effect size within regions of interest based on previous reports.** To compare cortical thinning reported in older cohorts with our younger cohort, we used Cohen’s *d* to examine differences between age-adjusted mean cortical thickness in ROIs highlighted in Rimol et al. (2010, 2012) between the bipolar disorder group (BPD; *n* = 73), psychosis group (PSY; *n* = 40) and controls (CON; *n* = 49). Regions of interest were defined by the Desikan–Killiany atlas (Desikan et al., 2006), where the inferior frontal gyrus (IFG) comprised the lateral and medial orbitofrontal regions, the middle frontal gyrus (MFG) comprised the rostral and caudal middle frontal regions, and the anterior cingulate cortex (ACC) comprised the rostral anterior and caudal anterior cingulate. ant., anterior; Lat Occip, lateral occipital gyrus; MTG, middle temporal gyrus; pos., posterior; SFG, superior frontal gyrus; STG, superior temporal gyrus; SPG, superior parietal gyrus.

|  | **Neurocognitive performance correlations (*r*/*rho*)** | | | | | | | | | | | | |
| --- | --- | --- | --- | --- | --- | --- | --- | --- | --- | --- | --- | --- | --- |
| **Regions of interest** | **RVP A'** | **RVP B"** | **RVP - mean latency** | **RAVLT - SUM** | **RAVLT - B1** | **RAVLT - A6** | **RAVLT - A7** | **COWAT - Letters** | **COWAT - Animals** | **TMT - A** | **TMT - B** | **PAL** | **IED ^#^** |
| ***Left hemisphere*** |  |  |  |  |  |  |  |  |  |  |  |  |  |
| Intraparietal sulcus | -0.25 | -0.14 | 0.01 | -0.08 | -0.07 | -0.04 | 0.09 | 0.05 | -0.08 | -0.27 | -0.09 | 0.04 | -0.41 |
| Angular gyrus (pos.) | -0.11 | 0.04 | -0.09 | 0.43 | **0.61^*^** | 0.48 | 0.55 | **0.63^*^** | **0.55^*^** | 0.14 | 0.07 | 0.36 | -0.06 |
| Angular gyrus (ant.) | 0.38 | 0.06 | **0.64^*^** | 0.30 | 0.17 | 0.35 | 0.46 | 0.34 | 0.37 | 0.06 | **0.61^*^** | 0.36 | -0.19 |
| Calcarine sulcus | -0.53 | -0.38 | 0.08 | 0.37 | 0.33 | 0.47 | 0.53 | 0.03 | 0.14 | -0.02 | -0.02 | -0.24 | -0.21 |
| ***Right hemisphere*** |  |  |  |  |  |  |  |  |  |  |  |  |  |
| Superior temporal gyrus | 0.27 | -0.28 | 0.19 | **0.65^*^** | 0.31 | **0.62^*^** | **0.69^**^** | **0.66^*^** | **0.67^*^** | 0.25 | 0.21 | 0.39 | 0.14 |
| Supramarginal gyrus | 0.35 | 0.07 | **0.72^**^** | 0.53 | **0.64^*^** | 0.55 | 0.55 | 0.37 | **0.56^*^** | 0.14 | 0.37 | 0.49 | -0.48 |
| Precuneus | 0.00 | 0.01 | 0.07 | 0.10 | -0.24 | 0.18 | 0.26 | -0.13 | -0.33 | -0.25 | 0.18 | -0.15 | -0.17 |
| Precentral gyrus | -0.22 | 0.32 | 0.16 | 0.21 | 0.00 | 0.14 | 0.29 | 0.23 | 0.06 | -0.12 | -0.01 | 0.14 | -0.19 |
| Fusiform | 0.13 | 0.26 | -0.04 | -0.13 | -0.29 | -0.11 | -0.19 | -0.22 | -0.19 | -0.16 | 0.17 | 0.02 | 0.32 |
| Parieto-occipical sulcus | -0.03 | -0.46 | -0.29 | 0.34 | -0.05 | 0.45 | 0.46 | 0.12 | -0.02 | -0.08 | -0.11 | -0.38 | -0.04 |
| Duration of illness | 0.32 | 0.31 | -0.19 | 0.10 | 0.28 | -0.03 | 0.02 | 0.16 | 0.34 | **0.63^*^** | 0.04 | 0.31 | -0.05 |

**Table 5. Interactions of cortical thinning or duration of illness with neurocognitive performance in subjects bipolar I disorder (*n* = 21).** Partial correlation examined the relationship between either cortical thickness in regions of interest or duration of illness with *z*-scores of neurocognitive performance, controlling for gender and years of education. # Spearman’s rho correlation analysis examined non-parametric distributions of *z*-scores. Significant associations were found between cortical thinning of the left posterior angular gyrus and neurocognitive deficits in verbal learning and verbal memory (RAVLT B1, *r* = 0.61, *n* = 15, *p* = .026), verbal fluency (COWAT Letters, *r* = 0.63, *n* = 15, *p* = .021); the left anterior angular gyrus and visual sustained attention (RVP mean latency, *r* = 0.64, *n* = 14, *p* = .026); the right superior temporal gyrus and verbal learning and verbal memory (RAVLT Sum, *r* = 0.65, *n* = 15, *p* = .016; RAVLT A6, *r* = 0.62, *n* = 15, *p* = .023; RAVLT A7, *r* = 0.69, *n* = 15, *p* = .010) and verbal fluency (COWAT Letters, *r* = 0.66, *n* = 15, *p* = .014; COWAT Animals, *r* = 0.67, *n* = 15, *p* = .012); and the right supramarginal gyrus and visual sustained attention (RVP mean latency, *r* = 0.72, *n* = 14, *p* = .008), verbal learning and verbal memory (RAVLT B1, *r* = 0.64, *n* = 15, *p* = .020) and verbal fluency (COWAT Animals, *r* = 0.56, *n* = 15, *p* = .045). Cortical thinning of the left anterior angular gyrus was associated with improvements in mental flexibility (TMT B, *r* = 0.61, *n* = 15, *p* = .027). Duration of illness was associated with reduced cognitive processing speed (TMT A, *r* = 0.63, *n* = 21, *p* = .017). * *p* < 0.05 (2-tailed), ** *p* < 0.01 (2-tailed). ant., anterior; COWAT, Controlled Word Association Test; IED, Intra/Extra-Dimensional shift; PAL, Paired Associate Learning; pos., posterior; RAVLT, Rey Auditory Verbal Learning Test; RVP, Rapid Visual Processing; TMT, Trail Making Test.

|  | **Neurocognitive performance correlations (*r*/*rho*)** | | | | | | | | | | | | |
| --- | --- | --- | --- | --- | --- | --- | --- | --- | --- | --- | --- | --- | --- |
| **Regions of interest** | **RVP A'** | **RVP B"** | **RVP - mean latency** | **RAVLT - SUM** | **RAVLT - B1** | **RAVLT - A6** | **RAVLT - A7** | **COWAT - Letters** | **COWAT - Animals** | **TMT - A** | **TMT - B** | **PAL ^#^** | **IED ^#^** |
| ***Left hemisphere*** |  |  |  |  |  |  |  |  |  |  |  |  |  |
| Intraparietal sulcus | 0.19 | -0.14 | -0.12 | 0.03 | 0.36 | 0.02 | 0.08 | 0.09 | 0.18 | 0.05 | 0.10 | 0.38 | -0.28 |
| Angular gyrus (pos.) | 0.19 | -0.06 | -0.25 | 0.14 | 0.16 | -0.01 | 0.10 | **-0.41^*^** | -0.32 | -0.05 | -0.11 | 0.01 | 0.04 |
| Angular gyrus (ant.) | 0.26 | -0.16 | 0.27 | -0.04 | -0.07 | 0.10 | 0.13 | 0.01 | 0.03 | -0.08 | -0.18 | 0.36 | -0.36 |
| Calcarine sulcus | 0.23 | 0.13 | 0.14 | 0.09 | 0.09 | 0.32 | 0.28 | -0.14 | 0.16 | 0.06 | -0.01 | **0.41^*^** | 0.04 |
| ***Right hemisphere*** |  |  |  |  |  |  |  |  |  |  |  |  |  |
| Superior temporal gyrus | 0.14 | **0.50^*^** | -0.07 | -0.06 | 0.08 | 0.13 | 0.21 | -0.14 | 0.12 | 0.08 | -0.12 | 0.15 | -0.15 |
| Supramarginal gyrus | 0.13 | 0.36 | 0.22 | -0.03 | 0.09 | 0.04 | 0.10 | 0.05 | 0.05 | 0.07 | -0.13 | 0.04 | -0.27 |
| Precuneus | 0.06 | 0.16 | -0.23 | 0.12 | 0.33 | 0.00 | 0.14 | **-0.58^**^** | -0.19 | 0.02 | -0.23 | -0.17 | 0.14 |
| Precentral gyrus | 0.27 | 0.27 | 0.18 | 0.06 | 0.12 | 0.06 | 0.10 | -0.12 | -0.26 | 0.02 | 0.14 | -0.04 | -0.11 |
| Fusiform | 0.14 | -0.20 | -0.09 | -0.12 | 0.09 | 0.07 | -0.04 | -0.03 | 0.01 | -0.04 | 0.21 | 0.16 | 0.10 |
| Parieto-occipical sulcus | 0.31 | -0.30 | 0.15 | 0.08 | -0.28 | 0.05 | 0.09 | -0.08 | 0.08 | -0.03 | -0.19 | 0.31 | -0.24 |
| Duration of illness | -0.31 | 0.11 | -0.05 | -0.31 | -0.10 | -0.28 | -0.31 | 0.25 | -0.21 | -0.38 | 0.00 | -0.23 | -0.18 |

**Table 6. Interactions of cortical thinning or duration of illness with neurocognitive performance in subjects bipolar II disorder (*n* = 29).** Partial correlation examined the relationship between either cortical thickness in regions of interest or duration of illness with *z*-scores of neurocognitive performance, controlling for gender and years of education. # Spearman’s rho correlation analysis examined non-parametric distributions of *z*-scores. Significant associations were found between cortical thinning of the right superior temporal gyrus and neurocognitive deficits in visual sustained attention (RVP B”, *r* = 0.50, *n* = 25, *p* = .014). Significant associations were found between cortical thinning of both the left posterior angular gyrus (COWAT Letters, *r* = -0.41, *n* = 27, *p* = .041) and right precuneus (COWAT Letters, *r* = -0.58, *n* = 27, *p* = .002) with improvements in verbal fluency. Finally cortical thinning of the right calcarine sulcus was associated with improvements in the episodic memory and learning task (PAL, *rho* = 0.41, *n* = 26, *p* = .036). * *p* < 0.05 (2-tailed), ** *p* < 0.01 (2-tailed). ant., anterior; COWAT, Controlled Word Association Test; IED, Intra/Extra-Dimensional shift; PAL, Paired Associate Learning; pos., posterior; RAVLT, Rey Auditory Verbal Learning Test; RVP, Rapid Visual Processing; TMT, Trail Making Test.

|  | **Neurocognitive performance correlations (*r*/*rho*)** | | | | | | | | | | | | |
| --- | --- | --- | --- | --- | --- | --- | --- | --- | --- | --- | --- | --- | --- |
| **Regions of interest** | **RVP A'** | **RVP B"** | **RVP - mean latency** | **RAVLT - SUM** | **RAVLT - B1** | **RAVLT - A6** | **RAVLT - A7** | **COWAT - Letters** | **COWAT - Animals** | **TMT - A** | **TMT - B** | **PAL ^#^** | **IED ^#^** |
| ***Left hemisphere*** |  |  |  |  |  |  |  |  |  |  |  |  |  |
| Intraparietal sulcus | -0.19 | 0.02 | **-0.62^*^** | 0.02 | 0.36 | -0.10 | -0.23 | 0.11 | -0.35 | -0.08 | -0.08 | 0.21 | -0.18 |
| Angular gyrus (pos.) | -0.30 | -0.49 | -0.40 | -0.01 | 0.01 | -0.06 | -0.33 | 0.18 | -0.02 | -0.26 | -0.33 | -0.34 | -0.20 |
| Angular gyrus (ant.) | 0.01 | 0.12 | -0.43 | 0.26 | 0.13 | 0.13 | 0.14 | 0.18 | -0.29 | -0.37 | -0.07 | 0.06 | -0.37 |
| Calcarine sulcus | 0.30 | 0.06 | -0.38 | -0.23 | -0.11 | -0.16 | -0.20 | 0.22 | -0.23 | -0.23 | 0.49 | 0.13 | -0.09 |
| ***Right hemisphere*** |  |  |  |  |  |  |  |  |  |  |  |  |  |
| Superior temporal gyrus | 0.20 | 0.14 | 0.38 | -0.01 | -0.36 | 0.07 | 0.21 | 0.36 | 0.16 | 0.16 | 0.17 | 0.05 | 0.02 |
| Supramarginal gyrus | 0.30 | -0.13 | 0.37 | 0.33 | -0.25 | 0.22 | 0.37 | -0.23 | 0.35 | -0.38 | 0.08 | 0.11 | -0.16 |
| Precuneus | -0.08 | -0.03 | -0.44 | 0.20 | -0.29 | 0.16 | 0.12 | 0.23 | -**0.49^*^** | -0.24 | 0.33 | -0.01 | -0.24 |
| Precentral gyrus | -0.43 | 0.14 | -0.43 | -0.01 | -0.31 | 0.15 | 0.07 | 0.21 | 0.20 | 0.06 | 0.22 | -0.33 | -0.04 |
| Fusiform | 0.26 | 0.03 | -0.04 | 0.45 | 0.18 | 0.30 | 0.28 | 0.45 | -0.45 | 0.15 | 0.30 | 0.41 | 0.11 |
| Parieto-occipical sulcus | -0.47 | 0.01 | -0.45 | -0.08 | -0.12 | -0.33 | -0.35 | -0.22 | 0.15 | **-0.79^**^** | -0.47 | **-0.50^*^** | **-0.84^**^** |
| Duration of illness | 0.07 | 0.17 | 0.23 | -0.23 | 0.34 | -0.26 | -0.28 | 0.08 | -0.25 | 0.31 | -0.40 | 0.13 | 0.14 |

**Table 7. Interactions of cortical thinning or duration of illness with neurocognitive performance in subjects with bipolar spectrum disorder (*n* = 23).** Partial correlation examined the relationship between either cortical thickness in regions of interest or duration of illness with *z*-scores of neurocognitive performance, controlling for gender and years of education. # Spearman’s rho correlation analysis examined non-parametric distributions of *z*-scores. Significant associations were found between cortical thinning of the left intraparietal sulcus and neurocognitive deficits in visual sustained attention (RVP mean latency, *r* = -0.62, *n* = 18, *p* = .010), and the right parieto-occipical sulcus and episodic memory and learning (PAL, *rho* = -0.50, *n* = 19, *p* = .030), mental flexibility (IED, *rho* = -0.84, *n* = 19, *p* < .001) and cognitive processing speed (TMT A, *r* = -0.79, *n* = 19, *p* < .001). There as a significant association between cortical thinning of the right precuneus and improved verbal fluency (COWAT Animals, *r* = -0.49, *n* = 19, *p* = .047). * *p* < 0.05 (2-tailed), ** *p* < 0.01 (2-tailed). ant., anterior; COWAT, Controlled Word Association Test; IED, Intra/Extra-Dimensional shift; PAL, Paired Associate Learning; pos., posterior; RAVLT, Rey Auditory Verbal Learning Test; RVP, Rapid Visual Processing; TMT, Trail Making Test.

|  | **Neurocognitive performance correlations (*r*/*rho*)** | | | | | | | | | | | | |
| --- | --- | --- | --- | --- | --- | --- | --- | --- | --- | --- | --- | --- | --- |
| **Regions of interest** | **RVP A'** | **RVP B"** | **RVP - mean latency** | **RAVLT - SUM** | **RAVLT - B1** | **RAVLT - A6** | **RAVLT - A7** | **COWAT - Letters** | **COWAT - Animals** | **TMT - A** | **TMT - B** | **PAL** | **IED** |
| ***Left hemisphere*** |  |  |  |  |  |  |  |  |  |  |  |  |  |
| Intraparietal sulcus | 0.19 | -0.31 | -0.24 | 0.03 | 0.20 | -0.03 | -0.02 | -0.18 | **-0.43^**^** | -0.17 | -0.18 | -0.12 | -0.01 |
| Angular gyrus (pos.) | 0.00 | -0.30 | 0.05 | 0.11 | 0.10 | 0.12 | 0.26 | 0.03 | -0.10 | -0.25 | 0.10 | 0.11 | 0.02 |
| Angular gyrus (ant.) | 0.13 | -0.22 | -0.20 | -0.12 | 0.07 | -0.13 | -0.08 | 0.03 | 0.08 | 0.03 | -0.19 | -0.08 | -0.08 |
| Calcarine sulcus | 0.17 | 0.19 | -0.04 | 0.27 | -0.10 | 0.29 | 0.20 | 0.15 | -0.07 | 0.20 | 0.24 | 0.15 | 0.29 |
| ***Right hemisphere*** |  |  |  |  |  |  |  |  |  |  |  |  |  |
| Superior temporal gyrus | 0.01 | -0.07 | 0.06 | -0.09 | -0.02 | -0.02 | -0.05 | 0.01 | -0.07 | -0.12 | 0.19 | -0.05 | -0.12 |
| Supramarginal gyrus | 0.12 | -0.01 | 0.32 | 0.07 | -0.20 | 0.05 | 0.10 | 0.07 | 0.08 | -0.06 | -0.24 | 0.20 | -0.07 |
| Precuneus | 0.20 | -0.24 | -0.06 | -0.13 | -0.19 | -0.15 | -0.16 | 0.08 | -0.15 | 0.01 | -0.03 | 0.08 | 0.07 |
| Precentral gyrus | -0.12 | -0.16 | **-0.36^*^** | -0.22 | -0.26 | -0.28 | -0.24 | **-0.44^**^** | **-0.48^**^** | -0.03 | **-0.38^*^** | -0.30 | -0.22 |
| Fusiform | 0.12 | -0.21 | 0.06 | 0.16 | 0.04 | 0.07 | 0.02 | -0.20 | **-0.35^*^** | -0.20 | -0.16 | 0.14 | 0.17 |
| Parieto-occipical sulcus | 0.19 | 0.12 | 0.15 | 0.19 | 0.21 | 0.18 | 0.12 | -0.26 | 0.23 | -0.07 | 0.04 | 0.10 | -0.03 |
| Duration of illness | 0.14 | 0.02 | 0.16 | 0.08 | -0.08 | 0.10 | 0.13 | 0.11 | 0.18 | -0.15 | -0.07 | -0.06 | 0.03 |

**Table 8. Interactions of cortical thinning or duration of illness with neurocognitive performance in subjects within the psychosis group (*n* = 40).** Partial correlation examined the relationship between either cortical thickness in regions of interest or duration of illness with *z*-scores of neurocognitive performance, controlling for gender and years of education. Significant associations were found between cortical thinning of the right precentral gyrus and neurocognitive deficits in visual sustained attention (RVP - mean latency, *r* = -0.36, *n* = 39, *p* = .029) and mental flexibility (TMT B, *r* = -0.38, *n* = 38, *p* = .037). Performance on the verbal fluency tests improved with cortical thinning to the left intraparital sulcus (COWAT Animals, *r* = -0.43, *n* = 37, *p* = .009), right precentral gyrus (COWAT Letters, *r* = -0.44, *n* = 38, *p* = .007; COWAT Animals, *r* = -0.48, *n* = 37, *p* = .003) and right fusiform (COWAT Animals, *r* = -0.35, *n* = 37, *p* = .042). * *p* < 0.05 (2-tailed), ** *p* < 0.01 (2-tailed). ant., anterior; COWAT, Controlled Word Association Test; IED, Intra/Extra-Dimensional shift; PAL, Paired Associate Learning; pos., posterior; RAVLT, Rey Auditory Verbal Learning Test; RVP, Rapid Visual Processing; TMT, Trail Making Test.

| **Partial correlation with medication dose, mg/day** | | | |
| --- | --- | --- | --- |
| **Region of interest** | **Antidepressants** | **Antipsychotics** | **Mood Stabilisers** |
| ***Left hemisphere*** |  |  |  |
| Intraparietal sulcus | -0.07 | 0.00 | -0.02 |
| Angular gyrus (pos.) | -0.22 | 0.18 | -0.04 |
| Angular gyrus (ant.) | -0.27 | 0.12 | 0.15 |
| Calcarine sulcus | -0.27 | 0.23 | 0.13 |
| ***Right hemisphere*** |  |  |  |
| Superior temporal gyrus | -0.21 | 0.02 | 0.06 |
| Supramarginal gyrus | -0.23 | -0.03 | 0.38 |
| Precuneus | -0.01 | 0.04 | -0.01 |
| Precentral sulcus | 0.17 | -0.07 | 0.50^**^ |
| Fusiform | 0.38^*^ | 0.07 | -0.04 |
| Parieto-occipical sulcus | -0.17 | 0.00 | -0.28 |
| Duration of illness | 0.03 | 0.24 | -0.42^*^ |

**Table 9. Interactions of cortical thinning or duration of illness with medication.** Partial correlation examined the relationship between either cortical thickness in regions of interest or duration of illness with classes of medication, controlling for gender and years of education. * Significance was set at *p* < .05 (two-tailed) ** *p* < .001 (two-tailed). ant., anterior; pos., posterior.
